# Principles of Hypotensive Shock: A Video Introduction to Pathophysiology and Treatment Strategies

**DOI:** 10.21980/J8MS84

**Published:** 2022-01-15

**Authors:** Brittany MacDonald, Nicholas MacDonald, Jacob Garcia, Xiao Chi Zhang, Dimitrios Papanagnou

**Affiliations:** * Sidney Kimmel Medical College, Thomas Jefferson University Hospitals, Philadelphia, PA; ^ Thomas Jefferson University Hospitals, Department of Emergency Medicine, Philadelphia, PA

## Abstract

**Audience:**

Emergency medicine interns, medical students, and mid-level providers (physician assistants, nurse practitioners).

**Introduction:**

Shock is defined as a state of global tissue hypoxia and is typically the result of hypotension and circulatory system failure. A variety of disease states may ultimately culminate in hypotensive shock through one or more generally recognized mechanisms – hypovolemic, cardiogenic, obstructive, and/or distributive shock.^1^ These mechanisms differ significantly in terms of their pathophysiology and requisite treatment. While the effects of hypotensive shock are initially reversible, untreated hypotensive shock may rapidly progress to multiorgan failure and death. Hence, the ability to promptly recognize a state of hypotensive shock, identify the underlying mechanism, and administer appropriate therapies are skills required of those caring for critically ill patients.^2^

The evaluation of hypotensive shock in the Emergency Department is relatively commonplace. Mortality rates associated with shock are high, ranging from 22.6% – 56.2%, depending upon the underlying etiology.^3^ For these reasons, the authors believe that a web-based learning module addressing topics related to hypotensive shock would be beneficial to healthcare professionals who are likely to encounter it in clinical practice. The web-based nature of the module would lend itself to convenient viewing and would allow for utilization as a just-in-time training modality. Presenting these topics in an animated format may also be a useful way of displaying the complex nature of cardiovascular physiology.

**Educational Objectives:**

By the end of this module, participants should be able to:

1. Review basic principles of cardiovascular physiology
2. Describe the four general pathophysiologic mechanisms of hypotensive shock
3. Recognize various etiologies for each mechanism of hypotensive shock
4. Recognize differences in the clinical presentation of each mechanism of hypotensive shock
5. Cite the basic approach to treatment for each mechanism of hypotensive shock

**Educational Methods:**

This is a video podcast which conveys information through animated content. It is available to learners on demand and just-in-time for practice. It may be used as a stand-alone educational tool, as a primer to other instructional methods (eg, simulation, flipped classroom setting, or case discussions), or a just-in-time training tool.

**Research Methods:**

A small-scale study was performed to quantify the efficacy of this module as an educational tool. The learner group was comprised of a convenience sample of third-year medical students in the midst of their core clinical clerkships. All third-year students were eligible to participate, regardless of which core clerkship they were currently engaged in. Third-year students were contacted via email regarding participation. Participation was completely optional – no incentive was offered, and students were informed that participation would not in any way affect their clerkship grades. For these reasons, an Instructional Review Board review was not necessary. Ten third-year medical students volunteered to participate. In the course of a single, hour-long session, learners were administered the attached assessment form as a pre-test, shown the video module, and then asked to immediately retake the assessment as a post-test to assess for improvement. Assessments were graded on a 17-point scale, according to the attached answer key. Learners were also given the opportunity to provide subjective feedback on the quality of the module as an educational tool.

**Results:**

For this assessment, the maximum possible score was 17 points. The average pre-test score across all learners was 11.75 (69.12%) with a standard deviation of 3.24. The average post-test score across all learners was 15.12 (88.97%) with a standard deviation of 3.31. All learners demonstrated improvement in scores on the post-test, with a maximum and minimum improvement of 6 points and 1 point respectively. On average, learners improved by 3.38 points (p = 0.029, 95% confidence interval, 1.97 to 4.78). Statistical significance was established using a paired student’s T-test. All learners agreed with the statement, “This module effectively taught concepts related to hypotensive shock.” Learners were also given the opportunity to provide subjective feedback regarding the module and responded with statements like, “comprehensive review of the subject,” and “very helpful review for clinical clerkships.”

**Discussion:**

Data from learner assessments suggest that this module is effective in communicating concepts related to hypotensive shock. Learner satisfaction with the module was unanimous. These results suggest that this module would be effective as a standalone educational tool, or as a primer to other instructional methods. Areas of further investigation may include assessment of a larger learner population, assessment of learners at additional stages of clinical training, and assessment of long-term knowledge retention.

**Topics:**

Shock, hypotension, cardiovascular physiology, pulmonary artery catheterization, flipped classroom, asynchronous learning, emergency medicine.

## USER GUIDE

[Table t1-jetem-7-1-l1]

**Table t1-jetem-7-1-l1:** 

List of Resources:	
Abstract	1
User Guide	3
Shock Video	4
Pre/Post-Test Questions	5
Pre/Post-Test Answers	8

**Learner Audience:**

Medical students, Interns, Mid-level providers (physician assistants, nurse practitioners)

**Time Required for Implementation:** 15 minutes

**Recommended Number of Learners per Instructor:**

This is a video podcast that can be viewed by learners on demand. It can be viewed by any number of learners, and no instructor is required. Instructors, however, have the opportunity to use this as preparatory material for a flipped classroom session or simulation.

**Topics:**

Shock, hypotension, cardiovascular physiology, pulmonary artery catheterization, flipped classroom, asynchronous learning, emergency medicine.

**Objectives:**

By the end of this module, participants should be able to:

1. Review basic principles of cardiovascular physiology
2. Describe the four general pathophysiologic mechanisms of hypotensive shock
3. Recognize various etiologies for each mechanism of hypotensive shock
4. Recognize differences in the clinical presentation of each mechanism of hypotensive shock
5. Cite the basic approach to treatment for each mechanism of hypotensive shock
6. Select standard diagnostic labs and tests commonly used in evaluating poisoned patients.

### Linked objectives and methods

This module instructs learners on cardiovascular physiology and the pathophysiology of hypotensive shock states. The animated nature of the module provides a unique mechanism for capturing the complexities and dynamic nature of this physiology. Additionally, the module is available to learners on demand, allowing for convenient viewing. It may be used as a stand-alone educational tool, as a primer to other instructional methods, or a just-in-time training tool.

### Link to Lecture

- https://youtu.be/tV-SSdWfEPw

### Recommended pre-reading for instructor

- Vincent JL, De Backer D. Circulatory shock. *N. Engl. J. Med*. 2013;369(18):1726–1734. doi:10.1056/NEJMra1208943.

### Results and tips for successful implementation

A small-scale study was performed to quantify the efficacy of this module as an educational tool. The learner group was comprised of a convenience sample of 10 third-year medical students in the midst of their core clinical clerkships. In the course of a single, hour-long session, learners were administered the attached assessment as a pre-test, shown the video module, and then asked to immediately retake the assessment as a post-test to assess for improvement. Assessments were graded on a 17-point scale, according to the attached answer key. Learners were also given the opportunity to provide subjective feedback on the quality of the module as an educational tool. The average pre-test score across all learners was 11.75 (69.12%) with a standard deviation of 3.24. The average post-test score across all learners was 15.12 (88.97%) with a standard deviation of 3.31. All learners demonstrated improvement in scores on the post-test, with a maximum and minimum improvement of 6 points and 1 point respectively. On average, learners improved by 3.38 points (p = 0.029, 95% confidence interval, 1.97 to 4.78). Statistical significance was established using a paired student’s T-test. All learners agreed with the statement, “This module effectively taught concepts related to hypotensive shock.” These results suggest that this module would be effective as a standalone educational tool, or as a primer to other instructional methods.

### Associated content (optional)

- Pre/Post Test
- Pre/Post Test Answers

### Technology necessary

Computer, tablet, or mobile device

## Figures and Tables

**Figure f1-jetem-7-1-l1:**
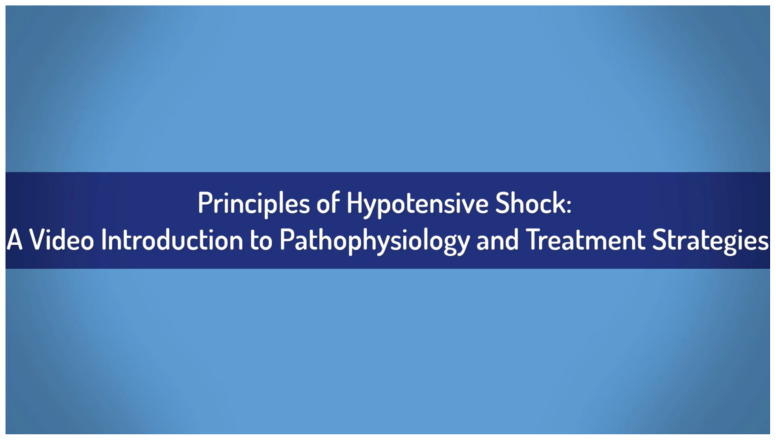
Please see associated video file Video Link: https://youtu.be/tV-SSdWfEPw

## LEARNER MATERIALS

### Shock Video

[Fig f1-jetem-7-1-l1]

### Pre/Post-Test Questions

#### MULTIPLE CHOICE QUESTIONS

1) Changes in which of the following parameters will have the most significant effect on vascular resistance?
  - Concentration of substances dissolved in blood
  - Temperature of blood
  - Vessel diameter
  - Vessel length
2) Which of the following is an appropriate indication for pulmonary artery catheterization?
  - Distributive shock that is refractory to vasopressor agents
  - Obstructive shock of any etiology
  - Patient meets criteria for Systemic Inflammatory Response Syndrome (SIRS)
  - Undifferentiated shock
3) Pulmonary capillary wedge pressure (PCWP) is typically used to approximate which of the following quantities?
  - Pressure within the left atrium
  - Pressure within the pulmonary arteries
  - Pressure within the right ventricle
  - Pulmonary vascular resistance
4) Which of the following represents the primary pathologic disturbance in cases of distributive shock?
  - Elevated cardiac output
  - Elevated systemic vascular resistance
  - Reduced cardiac output
  - Reduced systemic vascular resistance
5) Which of the following represents the primary pathologic disturbance in cases of hypovolemic shock?
  - Elevated cardiac output
  - Elevated systemic vascular resistance
  - Reduced cardiac output
  - Reduced systemic vascular resistance
6) If severe, pulmonary embolism has the potential to cause which type of hypotensive shock?
  - Cardiogenic
  - Distributive
  - Hypovolemic
  - Obstructive

#### Open-Ended Questions

7) Describe the mechanism of lactate elevation resulting from states of shock.
8) Cite 3 clinical findings that suggest a state of shock may be present in a given patient.
9) A patient with severe cardiogenic shock secondary to isolated left ventricular failure undergoes pulmonary artery catheterization. Compared to a healthy individual, what changes would you expect to see in this patient’s…
  - Cardiac output:
  - Systemic vascular resistance:
  - Pulmonary capillary wedge pressure:
10) Mean arterial blood pressure is a product of cardiac output and systemic vascular resistance (MAP = CO * SVR). For each of the following scenarios, what effect would the proposed change have on mean arterial blood pressure?
  - Increased heart rate:
  - Decreased myocardial contractility:
  - Increased venous return to the heart:
  - Decreased blood vessel diameter:

### Pre/Post-Test Answers

#### MULTIPLE CHOICE QUESTIONS

1) Changes in which of the following parameters will have the most significant effect on vascular resistance?
  - Concentration of substances dissolved in blood
  - Temperature of blood
  - **Vessel diameter (refer to 2:10 in the video)**
  - Vessel length
2) Which of the following is an appropriate indication for pulmonary artery catheterization?
  - Distributive shock that is refractory to vasopressor agents
  - Obstructive shock of any etiology
  - Patient meets criteria for Systemic Inflammatory Response Syndrome (SIRS)
  - **Undifferentiated shock (refer to 4:15 in the video)**
3) Pulmonary capillary wedge pressure (PCWP) is typically used to approximate which of the following quantities?
  - **Pressure within the left atrium (refer to 5:00 in the video)**
  - Pressure within the pulmonary arteries
  - Pressure within the right ventricle
  - Pulmonary vascular resistance
4) Which of the following represents the primary pathologic disturbance in cases of distributive shock?
  - Elevated cardiac output
  - Elevated systemic vascular resistance
  - Reduced cardiac output
  - **Reduced systemic vascular resistance (refer to 14:48 in the video)**
5) Which of the following represents the primary pathologic disturbance in cases of hypovolemic shock?
  - Elevated cardiac output
  - Elevated systemic vascular resistance
  - **Reduced cardiac output (refer to 6:45 in the video)**
  - Reduced systemic vascular resistance
6) If severe, pulmonary embolism has the potential to cause which type of hypotensive shock?
  - Cardiogenic
  - Distributive
  - Hypovolemic
  - **Obstructive (refer to 12:12 in the video)**

#### Open-Ended Questions

7) Describe the mechanism of serum lactate elevation resulting from states of shock.
  **Tissues that are poorly perfused become hypoxic. Now lacking oxygen, these tissues must resort to ANAEROBIC metabolism, a process that generates lactic acid as a biproduct. This lactic acid leads to elevated serum lactate levels, as well as an anion-gap metabolic acidosis. (refer to 3:50 in the video)**
8) Cite 3 clinical findings that suggest a state of shock may be present in a given patient.
  **Hypotension**
  **Tachycardia**
  **Tachypnea**
  **Oliguria**
  **Altered mental status**
  **Metabolic acidosis**
  **Elevated serum lactate**
  **(refer to 3:25 in the video)**
9) A patient with severe cardiogenic shock secondary to isolated left ventricular failure undergoes pulmonary artery catheterization. Compared to a healthy individual, what changes would you expect to see in this patient’s…
  - Cardiac output: **Decreased (primary pathologic insult)**
  - Systemic vascular resistance: **Increased (compensatory response)**
  - Pulmonary capillary wedge pressure: **Increased (primary pathologic insult) (refer to 9:15 in the video)**
10) Mean arterial blood pressure is a product of cardiac output and systemic vascular resistance (MAP = CO * SVR). For each of the following scenarios, what effect would the proposed change have on mean arterial blood pressure?
  - Increased heart rate: **Increased MAP**
  - Decreased myocardial contractility: **Decreased MAP**
  - Increased venous return to the heart: **Increased MAP**
  - Decreased blood vessel diameter: **Increased MAP (refer to 1:30 in the video)**
